# Anti-Müllerian hormone in PCOS: Molecular regulation and emerging therapeutic strategies

**DOI:** 10.17305/bb.2025.12070

**Published:** 2025-04-15

**Authors:** Yunmei Ke, Dan Tang, Qin Yang, Han Zhao, Jinyan Zheng, Caifen Zhu

**Affiliations:** 1 Department of Reproductive Gynecology, The First People’s Hospital of Yunnan Province, Kunming, Yunnan, China; 2 Department of Obstetrics and Gynecology, The Second People’s Hospital of Jinning District, Kunming, Yunnan, China; 3 Department of Gynecology, The Traditional Chinese Medicine Hospital of Jinning District, Kunming, Yunnan, China

**Keywords:** Polycystic ovary syndrome, PCOS, anti-Müllerian hormone, AMH, regulation, SMAD, therapy

## Abstract

Anti-Müllerian hormone (AMH), a glycoprotein belonging to the transforming growth factor-beta (TGF-β) superfamily, is a key regulator of ovarian folliculogenesis. Dysregulated AMH expression is a hallmark of polycystic ovary syndrome (PCOS), a common endocrine and metabolic disorder characterized by hyperandrogenism, anovulation, and polycystic ovarian morphology. Elevated AMH levels in PCOS impair follicle-stimulating hormone (FSH) sensitivity, disrupt follicular maturation, and contribute to androgen excess—creating a feedback loop that exacerbates ovarian dysfunction. This review explores the complex regulatory mechanisms governing AMH expression, including transcriptional, post-transcriptional, and post-translational processes. It highlights the interplay between AMH, FSH, and androgen signaling pathways, emphasizing their roles in the pathophysiology of PCOS. Particular attention is given to the downstream SMAD-dependent signaling cascade, which mediates many of AMH’s biological effects. Additionally, we summarize emerging therapeutic strategies targeting AMH signaling, such as anti-Müllerian hormone receptor type 2 (AMRH2) antagonists, gonadotropin-releasing hormone (GnRH) antagonists, and aromatase inhibitors. A deeper understanding of AMH regulation and signaling provides critical insights into its role in PCOS progression and supports the development of novel, targeted treatments aimed at alleviating both reproductive and metabolic symptoms.

## Introduction

**Figure 1.**
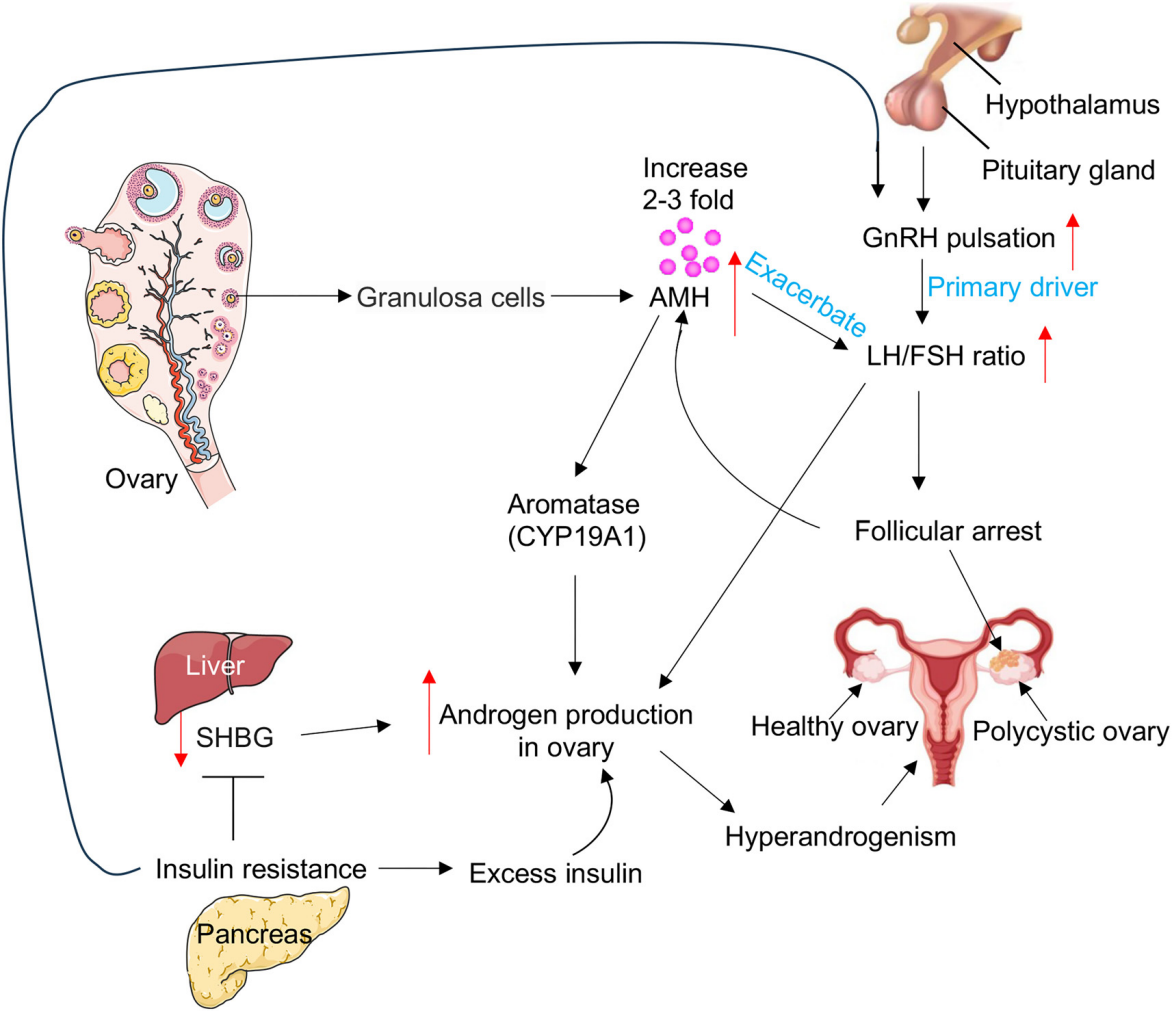
**Involvement of AMH, GnRH, LH/FSH, androgen, and insulin resistance in PCOS pathogenesis.** The pulsatile release of GnRH from the hypothalamus is often disturbed in PCOS, leading to an increased LH/FSH ratio. Elevated AMH further exacerbates this imbalance. Abnormal secretion of FSH and LH leads to follicular arrest, which in turn contributes to elevated AMH levels. Moreover, high AMH inhibits aromatase (CYP19A1) activity, leading to increased androgen production. The altered LH and FSH ratios impair ovulation: Elevated LH promotes hyperandrogenemia by stimulating androgen secretion from follicular theca cells, while reduced FSH levels contribute to anovulation. Additionally, insulin acts synergistically with LH to enhance androgen synthesis and decreases SHBG levels, thereby increasing the bioavailability of circulating androgens. Insulin resistance can also exacerbate PCOS by altering GnRH pulse frequency, affecting ovarian function, and increasing androgen levels. Abbreviations: GnRH: Gonadotropin-releasing hormone; SHBG: Sex hormone-binding globulin; LH: Luteinizing hormone; FSH: Follicle-stimulating hormone; AMH: Anti-Müllerian hormone; PCOS: Polycystic ovary syndrome.

Polycystic ovary syndrome (PCOS) is a complex endocrine disorder characterized by hyperandrogenism, ovulatory dysfunction, and polycystic ovarian morphology (PCOM). Its etiology is multifactorial, involving genetic, neuroendocrine, ovarian, and metabolic components [1–4]. A central aspect of PCOS pathogenesis is gonadotropin dysregulation, driven by an increased pulse frequency of gonadotropin-releasing hormone (GnRH), which preferentially stimulates the secretion of luteinizing hormone (LH) over follicle-stimulating hormone (FSH) (Figure 1) [5, 6]. The resulting elevated LH/FSH ratio promotes excess androgen production by theca cells, contributing to hyperandrogenism—a hallmark feature of PCOS (Figure 1) [5, 6].

Insulin resistance (IR) and hyperinsulinemia exacerbate the pathophysiology of PCOS by amplifying hyperandrogenism and contributing to metabolic dysfunction [7, 8]. Insulin acts synergistically with LH to stimulate androgen synthesis by theca cells and also reduces levels of sex hormone-binding globulin (SHBG), thereby increasing the bioavailability of circulating androgens (Figure 1) [7, 8]. Additionally, IR is strongly associated with metabolic disturbances, such as dyslipidemia, impaired glucose tolerance, and obesity—all of which further impair ovarian function [9, 10].

Another key contributor to PCOS is anti-Müllerian hormone (AMH), a member of the transforming growth factor β (TGF-β) superfamily, secreted by granulosa cells of pre-antral and small antral follicles [11–13]. In individuals with PCOS, AMH levels are abnormally elevated—typically two to three times higher than in healthy individuals—which further suppresses FSH sensitivity, contributing to follicular arrest and anovulation (Figure 1) [11–13]. AMH is also involved in neuroendocrine dysregulation, as it can enhance GnRH neuron activity, leading to increased LH secretion and perpetuating hormonal imbalances [11–13]. Moreover, elevated AMH levels inhibit aromatase (CYP19A1; cytochrome P450 family 19 subfamily A member 1) activity, resulting in androgen accumulation (Figure 1) [14]. This excess androgen production, driven by heightened LH stimulation of theca cells, further disrupts follicular development and ovulation [11–13].

Beyond its local effects on ovarian function, AMH may also exert systemic influences on the metabolic complications associated with PCOS [11–13]. Emerging evidence suggests that AMH plays a role in modulating insulin sensitivity, inflammatory signaling, lipid metabolism, and other metabolic processes [11–13, 15]. However, as the primary focus of this review is the regulatory mechanisms governing AMH expression in the pathogenesis of PCOS and its potential as a therapeutic target, we will not address its involvement in these additional biological processes.

## Regulatory mechanisms underlying AMH overexpression in PCOS

Elevated AMH expression is a defining characteristic of PCOS, and extensive research has explored its underlying regulatory mechanisms at multiple levels, including transcriptional, post-transcriptional, post-translational, and through crosstalk with other signaling pathways.

### AMH regulation at the transcriptional level

**Figure 2.**
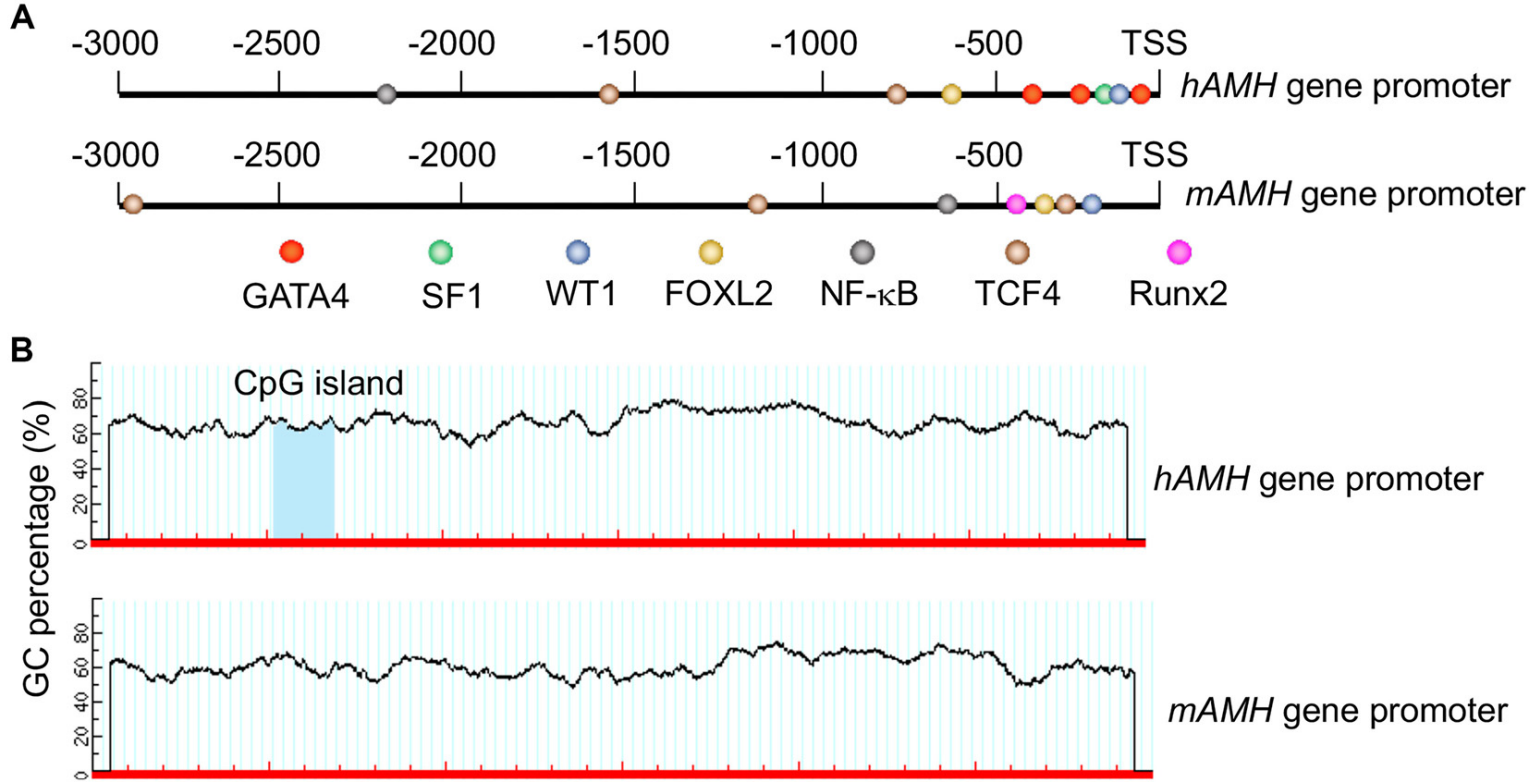
**Transcription factors and DNA methylation are involved in the transcriptional regulation of *AMH* gene.** (A) Transcription factors binding to the *hAMH* gene promoter (3000 bp upstream of the transcription start site, TSS) and the *mAMH* gene promoter and (B) GC content percentages in the *hAMH* and *mAMH* gene promoters, with the CpG island in the *hAMH* promoter highlighted. Abbreviations: AMH: Anti-Müllerian hormone; hAMH: Human AMH; mAMH: Mouse AMH.

The transcriptional regulation of *AMH* is orchestrated by a complex interplay of transcription factors and signaling pathways. These include GATA-binding factor 4 (GATA4) [16, 17], Steroidogenic factor 1 (SF1) [18], Forkhead box L2 (FOXL2) [19], and Wilms Tumor 1 (WT1) [20], which collectively ensure the precise control of AMH expression in granulosa cells (Figure 2A). In addition to these known transcription factors, our analysis revealed that the AMH promoter region contains binding sites for nuclear factor-kappa B (NF-κB), transcription factor 4 (TCF4), and runt-related transcription factor 2 (Runx2) (Figure 2A). However, no studies to date have provided evidence supporting their roles in regulating *AMH* gene expression.

#### The role of GATA4 in AMH gene expression

GATA4 is a transcription factor belonging to the GATA family, characterized by two zinc finger domains that are crucial for its function [21]. The C-terminal zinc finger is responsible for recognizing and binding to specific DNA sequences, while the N-terminal zinc finger stabilizes this interaction and facilitates protein–protein interactions with cofactors [21]. These structural features enable GATA4 to regulate the expression of key genes involved in gonadal development, sex determination, and steroidogenesis [16, 17].

During gonadal development in both mice and humans, GATA4 is prominently expressed in the somatic cells of the developing gonads, where it plays a pivotal role in regulating sex-determining genes, such as sex determining region Y (SRY) and SRY-box transcription factor 9 (SOX9), as well as genes involved in hormone production, including AMH, steroidogenic acute regulatory protein (STAR), and CYP19A1 [22]. GATA4 interacts cooperatively with transcriptional cofactors, such as SF1 and friend of GATA protein 2 (FOG2) to ensure the precise regulation of these genes [22]. Notably, GATA4 is essential for the activation of *AMH* during male embryonic development [23].

Experimental studies using CRISPR/Cas9-mediated inactivation of the GATA-binding motif in the *AMH* promoter provide direct evidence of GATA4’s role in regulating *AMH* expression [16]. In male fetal and neonatal testes, loss of GATA binding significantly reduced *AMH* mRNA and protein levels, although basal transcription was not entirely abolished [16]. This reduction impaired the expected upregulation of AMH during critical developmental windows. Despite the markedly lower AMH levels, they remained sufficient to permit normal male sexual differentiation, indicating that GATA4 functions in conjunction with other transcription factors to ensure adequate *AMH* expression during testis development [16, 17]. In contrast, in the adult ovary, GATA4 binding was found to be non-essential for maintaining AMH expression, suggesting that its regulatory role is both tissue- and stage-specific [16, 17].

Additionally, studies in ovarian granulosa cells highlight GATA4’s critical role in AMH transcription [24]. By binding to conserved promoter sequences, GATA4 enhances *AMH* gene expression and operates synergistically with other factors, such as FOXL2 and SF1, forming a complex transcriptional regulatory network [24]. These interactions ensure robust control of *AMH* expression, which is further modulated by signaling pathways such as those mediated by gonadotropins [24]. This dynamic regulation reflects the hormonal and developmental contexts in which GATA4 functions, underscoring its importance in both testicular and ovarian physiology [24].

#### Cyclic AMP (cAMP) and SF1-associated transcriptional networks

Although classical cAMP-protein kinase A (PKA) signaling is known to increase AMH expression, studies have shown that the *AMH* promoter lacks a canonical cAMP response element (CRE), suggesting the involvement of alternative pathways and transcription factors [25]. The *AMH* promoter contains binding sites for SOX9, SF1, GATA4, and Activating protein 1 (AP1), all of which are implicated in cAMP-responsive gene regulation [25]. Experimental studies using Sertoli cells have demonstrated that these factors mediate cAMP-induced *AMH* transcription [26, 27]. Beyond the classical PKA pathway, additional cAMP-regulated cascades—including the cAMP-Guanine nucleotide exchange factor–Phosphatidylinositol 3-kinase–Protein kinase B (GEF-PI3K-Akt) pathway and mitogen-activated protein kinase (MAPK) signaling—also enhance *AMH* promoter activity [28].

Among the transcription factors involved in AMH regulation, SOX9 and SF1 play pivotal roles [29]. SF1 binds to a key element in the *AMH* promoter and cooperates with SOX9—a critical regulator of Sertoli cell differentiation—to amplify *AMH* transcription [29]. Protein-binding studies have shown that SOX9 and SF1 form a functional complex through interactions between their DNA-binding and C-terminal regions, respectively [29]. This combinatorial mechanism ensures cell- and stage-specific AMH expression during embryogenesis, underscoring the intricate transcriptional and signaling network required for proper male sexual differentiation [29].

#### FBXL12’s roles in the regulation of AMH expression

FOXL2 is a transcription factor essential for ovarian development and function. It directly binds to the *AMH* promoter and interacts with other transcription factors, such as SF1, to regulate *AMH* expression [30]. This interaction forms a transcriptional complex that enhances *AMH* promoter activity, ensuring proper ovarian function. In addition to its role in transcriptional regulation, FOXL2 protects granulosa cells from apoptosis, thereby supporting AMH production by maintaining cell viability [30]. Mutations in FOXL2—observed in conditions, such as blepharophimosis, ptosis, epicanthus inversus syndrome (BPES) and certain granulosa cell tumors—are associated with dysregulated AMH expression, highlighting FOXL2’s critical role in ovarian health [31].

Recent studies indicate that AMH can upregulate both the gene and protein expression of FOXL2 [32], suggesting the existence of a positive feedback loop that helps preserve the ovarian follicle reserve. *In vivo* experiments have shown that knocking down AMH accelerates follicle growth—an effect that can be mitigated by ectopic expression of FOXL2 [19]. This highlights the coordinated interplay between FOXL2 and AMH in regulating ovarian follicle development [19]. Functional FOXL2 is also essential for SF1-induced AMH regulation, as it facilitates the association between SF1 and the *AMH* promoter [19]. Mutations in FOXL2 disrupt this interaction, leading to impaired *AMH* expression and subsequent ovarian dysfunction [19].

#### WT1’s influence on AMH transcription

The WT1 transcription factor is essential for mammalian urogenital development, playing a central role in both gonadal differentiation and Müllerian duct regression [33]. Mutations in the WT1 gene are linked to several disorders, including Wilms’ tumor—a pediatric kidney cancer—as well as syndromes, such as Denys–Drash and Frasier [33]. In severe cases of Denys–Drash syndrome, affected individuals may exhibit pseudohermaphroditism or complete sex reversal. During sexual development, WT1 regulates key genes, including anti-Müllerian hormone receptor type 2 (AMHR2) [33]. AMHR2 is critical for Müllerian duct regression in males, and mutations in this gene lead to persistent Müllerian duct syndrome, a rare condition characterized by male pseudohermaphroditism [33]. WT1 and AMHR2 are coexpressed during urogenital development, with WT1 directly binding to the *AMHR2* promoter to regulate its transcription [33]. Experimental models show that changes in WT1 expression cause immediate alterations in *AMHR2* levels, underscoring WT1’s role as a key regulator of Müllerian duct regression [33].

In addition to its role in regulating AMHR2, WT1 also influences *AMH* expression. In fetal Sertoli cells, WT1 binds directly to the *AMH* promoter and interacts with transcription factors, such as SF1 and GATA4 to regulate basal *AMH* transcription, ensuring adequate expression for Müllerian duct regression [34]. However, WT1’s function is not limited to male development. In females, WT1 is expressed in granulosa cells, where it modulates *AMH* levels within the ovarian microenvironment [35]. This highlights WT1’s regulatory role as both context- and tissue-specific, shaped by co-regulatory proteins and the cellular environment. While its involvement in male sexual differentiation is well-established, further research is needed to clarify WT1’s precise mechanisms in ovarian granulosa cells and its broader role in ovarian physiology [34, 35].

#### DNA methylation in the regulation of AMH

DNA methylation is a key epigenetic mechanism that regulates gene expression by adding methyl groups to cytosine residues within CpG dinucleotides, particularly in promoter regions [36]. This modification, catalyzed by DNA methyltransferases (DNMTs), alters chromatin structure and inhibits the binding of transcription factors and RNA polymerase, ultimately leading to gene silencing [36].

We analyzed the GC content and CpG islands in the promoter regions (3000 bp upstream of the transcription start site) of the human and mouse AMH genes. The analysis revealed that the mouse AMH promoter has a GC content of 60.9%, with no CpG islands identified. In contrast, the human AMH promoter exhibits a higher GC content of 66.47% and contains a CpG island (Figure 2B). Several studies have highlighted the role of DNA methylation in regulating *AMH* gene expression. For example, research on multiple sclerosis (MS) patients demonstrated that increased methylation of the *AMH* promoter correlates with reduced gene expression, linking methylation to disease activity [37]. Similarly, studies of children born to women with PCOS revealed altered methylation patterns in genes associated with reproductive function, including AMH, suggesting potential effects on ovarian follicle development [38]. Additional research in ovarian granulosa cells from PCOS patients showed that aberrant methylation of the *AMH* promoter disrupts gene expression, contributing to the hormonal imbalances characteristic of the condition [38]. Collectively, these findings demonstrate that DNA methylation in the *AMH* promoter region is a pivotal regulatory mechanism influencing gene expression.

### AMH regulation at the post-transcriptional level

Currently, two major mechanisms of post-transcriptional regulation, microRNAs (miRNAs) and long non-coding RNAs (lncRNAs), play critical roles in regulating *AMH* expression.

#### miRNAs involved in the regulation of AMH expression

miRNAs are small, non-coding RNA molecules approximately 22 nucleotides in length that play a pivotal role in post-transcriptional gene regulation [39]. They function by binding to complementary sequences on target messenger RNAs (mRNAs), leading to mRNA degradation or inhibition of translation, thereby modulating gene expression [39].

Recent studies have identified specific miRNAs that directly target the *AMH* gene, thereby influencing its expression. For example, the miR-200 family—including miR-200a, miR-200b, miR-200c, miR-141, and miR-429—has been shown to regulate *AMH* expression in Japanese flounder (Paralichthys olivaceus), affecting gonadal development [40]. These findings suggest that miR-200 family members may modulate *AMH* levels and contribute to reproductive processes. Similarly, miR-140-3p has been demonstrated to promote the proliferation of follicular granulosa cells and enhance steroid hormone synthesis by directly targeting the *AMH* gene in chickens [41]. This interaction downregulates *AMH* expression, thereby boosting granulosa cell activity and hormone production—both critical for follicular development and ovulation [41]. Additionally, miR-155 has been identified as a potential biomarker of subfertility in men with chronic kidney disease. Although a direct interaction between miR-155 and *AMH* has not been established, the observed correlation suggests it may indirectly affect *AMH* expression and influence male fertility [42].

**Figure 3.**
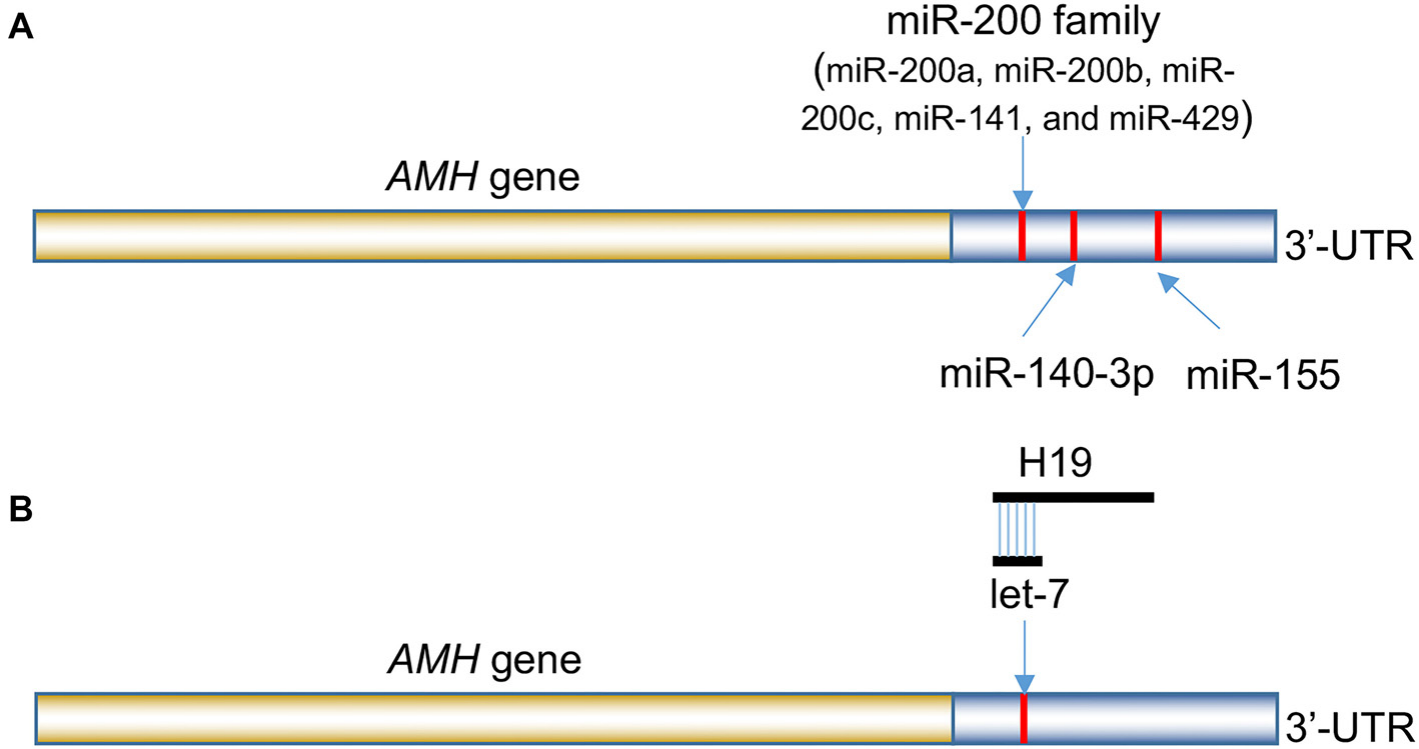
**miRNAs and the HT19 lncRNA are involved in the regulation of the *AMH* gene at the post-transcriptional level.** (A) The miR-200 family, miR-140-3p, and miR-155 target the 3’-UTR of *AMH* genes across various species and (B) The H19 lncRNA indirectly regulates *AMH* gene expression by acting as a molecular “sponge” for let-7, which targets the 3’-UTR of *AMH*. Abbreviations: AMH: Anti-Müllerian hormone; miRNA: Micro RNA; lncRNA: Long non-coding RNA.

These findings underscore the complex regulatory networks involving miRNAs that control *AMH* expression. By binding to specific sites on AMH mRNA, miRNAs, such as the miR-200 family, miR-140-3p, and miR-155 can modulate mRNA stability and translation efficiency, thereby influencing reproductive development and function (Figure 3A). Understanding these mechanisms offers valuable insights into the post-transcriptional regulation of AMH and its broader implications for reproductive health.

#### H19 in the regulation of AMH expression

lncRNAs are RNA molecules longer than 200 nucleotides that do not encode proteins but serve as essential regulators of gene expression [43]. At the post-transcriptional level, lncRNAs can interact with mRNAs to influence their splicing, stability, and translation efficiency [43]. Additionally, they can act as molecular sponges for miRNAs, sequestering them and preventing their interaction with target mRNAs, thereby indirectly regulating gene expression [43]. One such lncRNA, H19, has emerged as a key player in reproductive biology, particularly in the regulation of ovarian function [44]. The ovarian reserve—comprising follicles and oocytes—declines with age, leading to reduced fertility. Women with diminished ovarian reserve (DOR) show lower circulating and ovarian H19 levels, which are associated with decreased serum AMH levels [44]. Studies in H19 knockout (H19KO) mice reveal phenotypes resembling those of AMH knockout (AMHKO) mice, including accelerated follicular recruitment, subfertility, and reduced *AMH* mRNA and protein expression [45]. Notably, *AMH* mRNA contains a functional Let7 miRNA binding site, suggesting that H19 may regulate *AMH* expression via the Let7 pathway. In H19KO mice, superovulation results in increased estradiol production and oocyte yield, indicating that H19 acts to limit the number of ovulating follicles [44, 45]. Collectively, these findings highlight H19’s critical role in modulating *AMH* expression and maintaining the ovarian reserve, likely through its interaction with Let7 (Figure 3B).

### AMH expression regulation at the post-translational level

**Figure 4.**
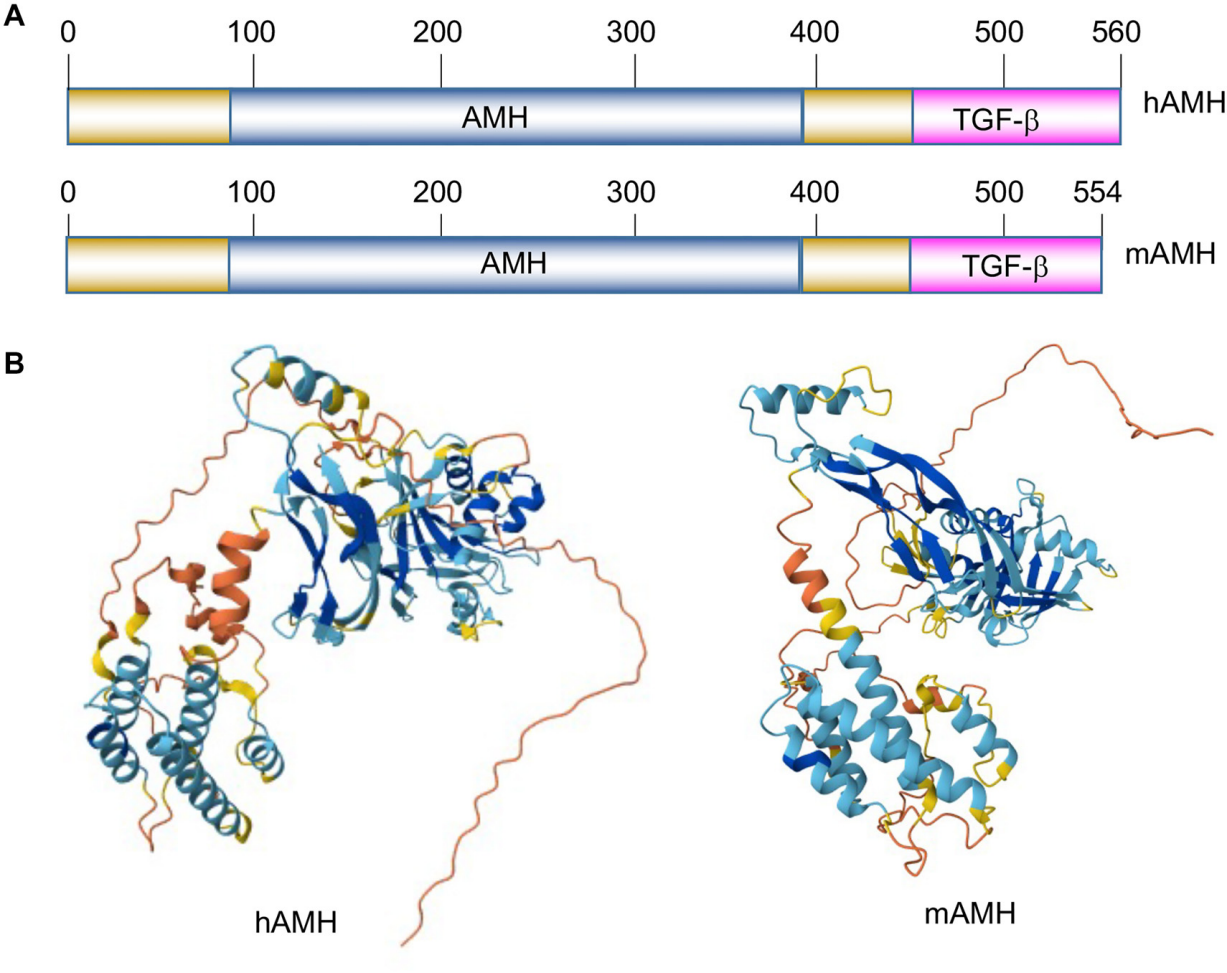
**Functional domains and structures of AMH proteins.** (A) Conserved domains of hAMH and mAMH proteins, highlighting two main functional domains: The AMH-specific domain and the conserved TGF-β family domain and (B) Predicted three-dimensional structures of hAMH and mAMH generated by AlphaFold. Abbreviations: AMH: Anti-Müllerian hormone; TGF-β: Transforming growth factor β; hAMH: Human AMH; mAMH: Mouse AMH.

The AMH protein in both humans and mice contains two conserved functional domains: the AMH domain and the TGF-β family domain (Figure 4A) [46]. Three-dimensional structural predictions generated using AlphaFold reveal a high degree of similarity between the human and mouse AMH proteins (Figure 4B), highlighting the evolutionary conservation of their functional architecture. Post-translational mechanisms—such as proteolytic cleavage, glycosylation, dimerization, and interaction with the prodomain—also play critical roles in regulating AMH activity. These modifications ensure proper processing, stability, and biological activity of AMH, enabling it to carry out its essential functions in reproductive development and regulation.

#### Proteolytic cleavage

AMH is initially synthesized as an inactive precursor—a 140 kDa homodimer composed of a 72 kDa N-terminal pro-region and a 25 kDa C-terminal mature region [10]. To become biologically active, this precursor must undergo proteolytic cleavage at specific sites, primarily between arginine and serine residues at positions 427 and 428 [47]. This cleavage separates the pro-region from the mature domain, a necessary step for AMH to exert its biological functions [47]. The process is mediated by subtilisin-like proprotein convertases, such as furin [48]. Mutations that disrupt these cleavage sites can lead to reduced or absent AMH activity, impairing its role in Müllerian duct regression during male embryogenesis and in follicular regulation in females [48]. Studies have shown that the efficiency of this cleavage process directly impacts the amount of active AMH available, underscoring its critical role in AMH functionality [49, 50]. For instance, mice harboring mutations that impair cleavage display phenotypes consistent with AMH deficiency, including persistent Müllerian structures in males and disrupted folliculogenesis in females [49, 50].

#### Glycosylation

AMH is a glycoprotein, and its glycosylation is essential for proper folding, stability, and secretion. This modification occurs in the endoplasmic reticulum and Golgi apparatus during protein synthesis, where specific carbohydrate moieties are added to asparagine residues on the AMH molecule [51, 52]. Glycosylation enhances the solubility and extracellular stability of AMH while protecting it from proteolytic degradation. It also facilitates efficient secretion from Sertoli cells in males and granulosa cells in females [51, 52]. Moreover, variations in glycosylation patterns can influence AMH’s receptor-binding affinity, thereby affecting its interaction with AMHR2 and the initiation of downstream signaling [51, 52]. Aberrant glycosylation has been linked to reduced AMH bioactivity and may contribute to reproductive disorders such as PCOS or DOR [51, 52]. Understanding the specific glycosylation patterns of AMH could provide valuable insights into its regulation and functional role in reproductive health.

#### Dimerization

AMH functions as a disulfide-linked homodimer, a configuration essential for its structural stability and receptor-binding affinity [47, 53]. This dimer forms in the endoplasmic reticulum during protein synthesis, where disulfide bonds are established between two identical monomers [47, 53]. Dimerization ensures the correct spatial arrangement of AMH, which is crucial for its interaction with the AMH receptor, AMHR2 [47, 53]. The dimeric structure enables AMH to bind AMHR2 with high specificity and activate intracellular signaling pathways, including the SMAD-dependent cascade that regulates gene expression involved in reproductive processes [47, 53]. Defects in dimerization can result in misfolded or biologically inactive AMH. Studies using recombinant AMH mutants with disrupted dimerization have demonstrated reduced receptor binding and impaired signaling, underscoring the critical role of this structural modification in AMH function [47, 53].

#### Interaction with prodomain

Even after proteolytic cleavage, the N-terminal prodomain remains non-covalently associated with the C-terminal mature domain of AMH [54, 55]. This interaction is critical for maintaining the structural stability and functional integrity of AMH in the extracellular environment. Acting as a molecular chaperone, the prodomain stabilizes the mature domain and protects it from premature degradation or denaturation [54, 55]. Importantly, this association also prevents AMH from interacting with its receptor, AMHR2, until the prodomain dissociates—ensuring that AMH activity is spatially and temporally regulated [54, 55]. Upon receptor engagement, the prodomain separates from the mature domain, allowing it to bind AMHR2 and initiate downstream signaling pathways [54, 55]. This regulatory mechanism provides precise control over AMH activity, preventing inappropriate or excessive signaling that could interfere with reproductive development and function.

### Regulation of AMH by signaling pathways

AMH plays a pivotal role in reproductive signaling pathways. Upon binding to its receptor, AMHR2, AMH activates the SMAD-dependent signaling cascade, which modulates gene expression essential for ovarian folliculogenesis and the maintenance of the ovarian reserve [46, 55]. By inhibiting primordial follicle activation and decreasing FSH sensitivity in developing follicles, AMH ensures controlled and gradual follicular growth. Dysregulation of AMH signaling—particularly within the SMAD pathway—is implicated in reproductive disorders such as PCOS, where elevated AMH levels disrupt follicular dynamics and contribute to hormonal imbalances [46, 55].

#### AMH and SMAD-dependent signaling pathway

**Figure 5.**
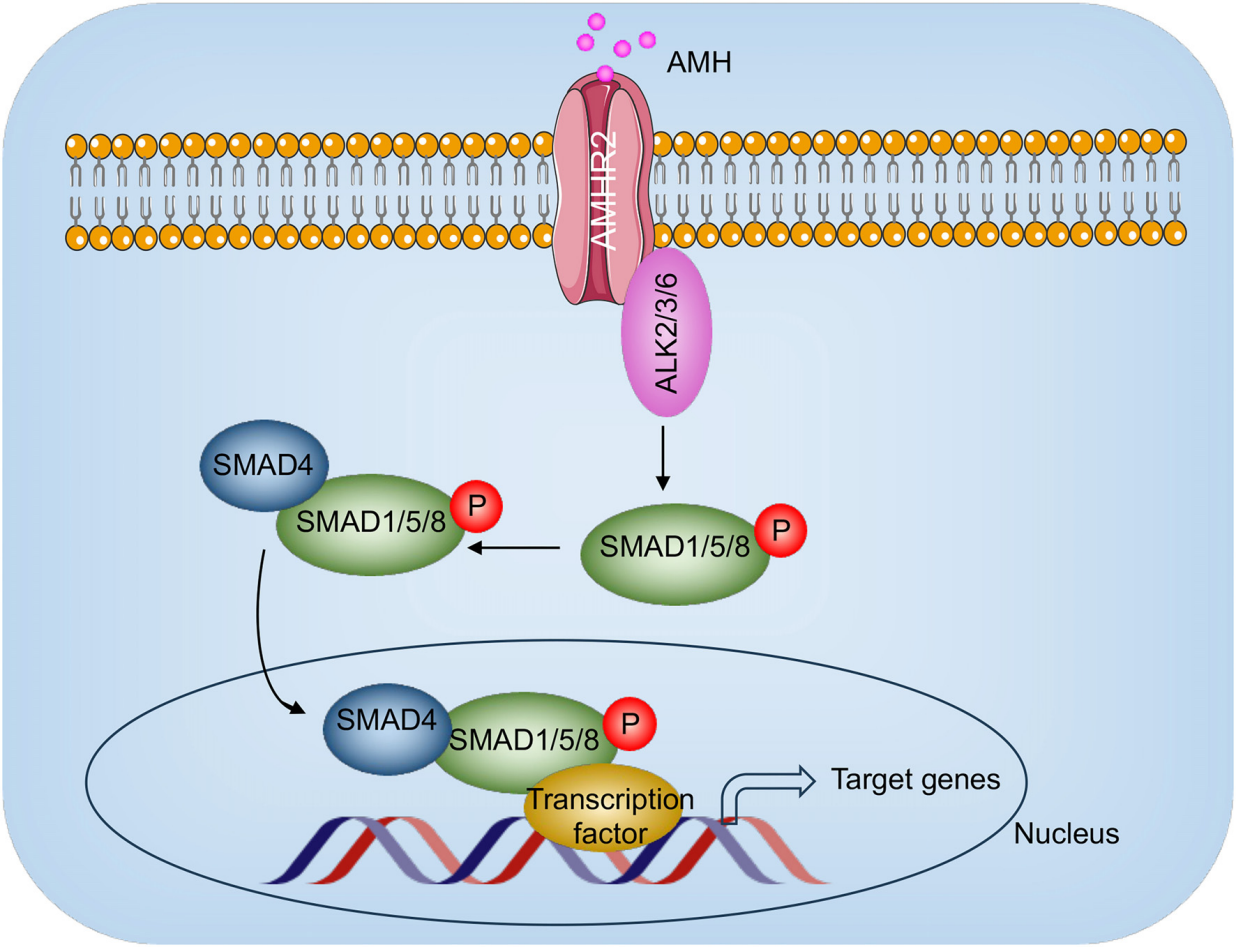
**AMH/AMHR2 signaling pathway in the pathogenesis of PCOS.** AMH binds to its receptor AMHR2, which interacts with ALK2/3/6 to activate SMAD proteins 1/5/8. Phosphorylated SMAD1/5/8 translocate to the nucleus alongside SMAD4, where they cooperate with transcription factors (TFs) to regulate the expression of AMH target genes. Abbreviations: PCOS: Polycystic ovary syndrome; AMH: Anti-Müllerian hormone; AMHR2: Anti-Müllerian hormone receptor type 2.

The SMAD-dependent signaling pathway is a key mechanism by which members of the TGF-β superfamily, including AMH, regulate cellular processes, such as growth, differentiation, and development [46, 55]. Upon binding to AMHR2, AMH triggers the formation of a receptor complex that recruits and phosphorylates receptor-regulated SMADs (R-SMADs), specifically SMAD1, SMAD5, and SMAD8 [46, 55]. These phosphorylated R-SMADs then associate with the common mediator SMAD4 and translocate into the nucleus. Once inside the nucleus, the SMAD complex interacts with transcription factors and co-regulators to modulate the expression of target genes (Figure 5) [46, 55]. These genes play critical roles in ovarian follicular development, including primordial follicle recruitment and granulosa cell differentiation, highlighting the central role of SMAD signaling in reproductive health.

AMH plays a key role in folliculogenesis by modulating SMAD-dependent signaling to regulate the recruitment and growth of ovarian follicles [56]. Specifically, AMH inhibits the initial activation of primordial follicles, thereby preserving the ovarian reserve and ensuring a sustained supply of oocytes throughout reproductive life [57]. It also reduces FSH sensitivity in growing follicles, helping to control the pace of follicular development. Dysregulation of AMH expression or disruptions in SMAD signaling can impair ovarian function and contribute to the development of reproductive disorders [57].

In the context of PCOS, elevated AMH levels are a hallmark feature and play a significant role in disease pathophysiology. Excess AMH leads to heightened activation of the SMAD1/5/8 signaling pathway, which intensifies the inhibition of follicular recruitment and reduces follicle sensitivity to FSH [58]. This contributes to the accumulation of small antral follicles and the follicular arrest characteristic of PCOS. Additionally, high AMH levels impact granulosa cell function, promoting hyperandrogenism and creating a feedback loop that further exacerbates hormonal imbalances [58]. This dysregulation of the AMH–SMAD signaling axis not only impairs normal follicular development but also disrupts endocrine signaling, amplifying both the reproductive and metabolic disturbances associated with PCOS [58].

#### Interplay between AMH and FSH

The interaction between AMH and FSH is essential for regulating ovarian folliculogenesis and maintaining reproductive health [59, 60]. AMH inhibits the recruitment of primordial follicles into the growing follicle pool, thereby preserving the ovarian reserve and ensuring a consistent supply of oocytes throughout a woman’s reproductive lifespan [59, 60]. In contrast, FSH promotes the growth and maturation of follicles, serving as the primary driver for the selection of a dominant follicle during each menstrual cycle. The delicate balance between AMH and FSH is critical for normal ovarian function and reproductive homeostasis [59, 60].

AMH modulates FSH activity by reducing the sensitivity of granulosa cells to FSH in developing follicles [61]. This regulation prevents premature follicular recruitment and overactivation, ensuring that only follicles with sufficient FSH receptor expression progress to dominance [61]. Research has shown that AMH suppresses FSH-induced aromatase expression, thereby lowering estradiol production in granulosa cells. By controlling FSH responsiveness, AMH fine-tunes the transition of follicles through critical developmental stages and plays a key role in maintaining balanced ovarian function [61].

In PCOS, the interplay between AMH and FSH is significantly disrupted. Women with PCOS typically exhibit elevated AMH levels, which further reduce FSH sensitivity in granulosa cells. This heightened FSH insensitivity contributes to follicular arrest, where small antral follicles accumulate but fail to progress to the dominant stage, resulting in anovulation [58, 59]. Additionally, elevated AMH is believed to suppress FSH-mediated granulosa cell differentiation, exacerbating the hormonal imbalance that characterizes PCOS [58, 59].

#### Androgens and their role in AMH regulation

Androgens, such as testosterone and dihydrotestosterone (DHT), play a significant role in regulating AMH expression within the ovary [59, 62]. Granulosa cells, the primary source of AMH, express androgen receptors (ARs), allowing them to respond directly to androgenic signals—an interaction that is essential for normal folliculogenesis and ovarian functions. Studies have shown that androgens can modulate AMH levels in granulosa cells [59, 62]. For example, exposure to DHT has been associated with increased AMH production, indicating a stimulatory effect mediated through the AR pathway [63]. This highlights the direct influence of androgens on granulosa cell function. In the context of PCOS, a condition marked by hyperandrogenism, elevated androgen levels contribute to increased AMH expression [64]. This elevation is linked to disrupted follicular development and anovulation—hallmark features of PCOS. High AMH levels in this setting may further inhibit FSH sensitivity, impairing follicle maturation and contributing to ovulatory dysfunction [64].

## Therapeutic potentials targeting AMH in PCOS

Given the critical role of AMH in the pathophysiology of PCOS—particularly its involvement in disrupting folliculogenesis and contributing to anovulation—targeting AMH represents a promising therapeutic strategy. Emerging approaches aim to modulate AMH signaling or reduce its overexpression in order to restore normal ovarian function and improve fertility outcomes in affected individuals [65].

### AMH receptor antagonists

Developing antagonists that block AMH binding to its receptor, AMHR2, represents a direct strategy to mitigate excessive AMH signaling. AMHR2 antagonists are gaining traction as promising therapeutic agents across various fields, particularly in oncology. By inhibiting AMHR2-mediated signaling, these antagonists can suppress tumor growth in cancers that express this receptor [66]. One example is murlentamab, a humanized monoclonal antibody developed by LFB Biotechnologies, which targets AMHR2 and is currently under investigation for its efficacy against multiple tumor types [66]. In addition, several AMHR2 agonists—such as SP600125, CYC-116, gandotinib, and ruxolitinib—have been shown to repress folliculogenesis in mice and rats [67]. Gandotinib has been evaluated in a Phase II clinical trial (NCT01594723) for myeloproliferative disorders, including myelofibrosis, polycythemia vera, and essential thrombocythemia [67, 68]. Ruxolitinib has received regulatory approval for the treatment of intermediate- to high-risk myelofibrosis, polycythemia vera (in patients unresponsive or intolerant to hydroxyurea), and graft-versus-host disease [67, 69]. Another promising agent is GM102, a humanized, glyco-engineered monoclonal antibody targeting AMHR2. Following extensive pharmacological characterization and toxicology studies in cynomolgus monkeys, GM102 entered a Phase I clinical trial in patients with gynecologic malignancies (NCT02978755) [70]. In the context of PCOS, AMHR2 antagonists have been proposed as a novel therapeutic approach aimed at restoring normal folliculogenesis and ovulation by counteracting elevated AMH levels. While this concept shows promise, it remains largely theoretical, and clinical trials are needed to assess the safety and efficacy of AMHR2 antagonists in treating PCOS [71]. Additionally, a monoclonal antibody targeting AMH itself—referred to as B10—has demonstrated the ability to reduce cell viability and induce apoptosis in four ovarian cancer cell lines, as well as in ascitic cells derived from ovarian cancer patients [72]. These findings suggest its potential utility as a therapeutic agent not only in oncology but possibly in conditions like PCOS as well.

### GnRH antagonists

**Figure 6.**
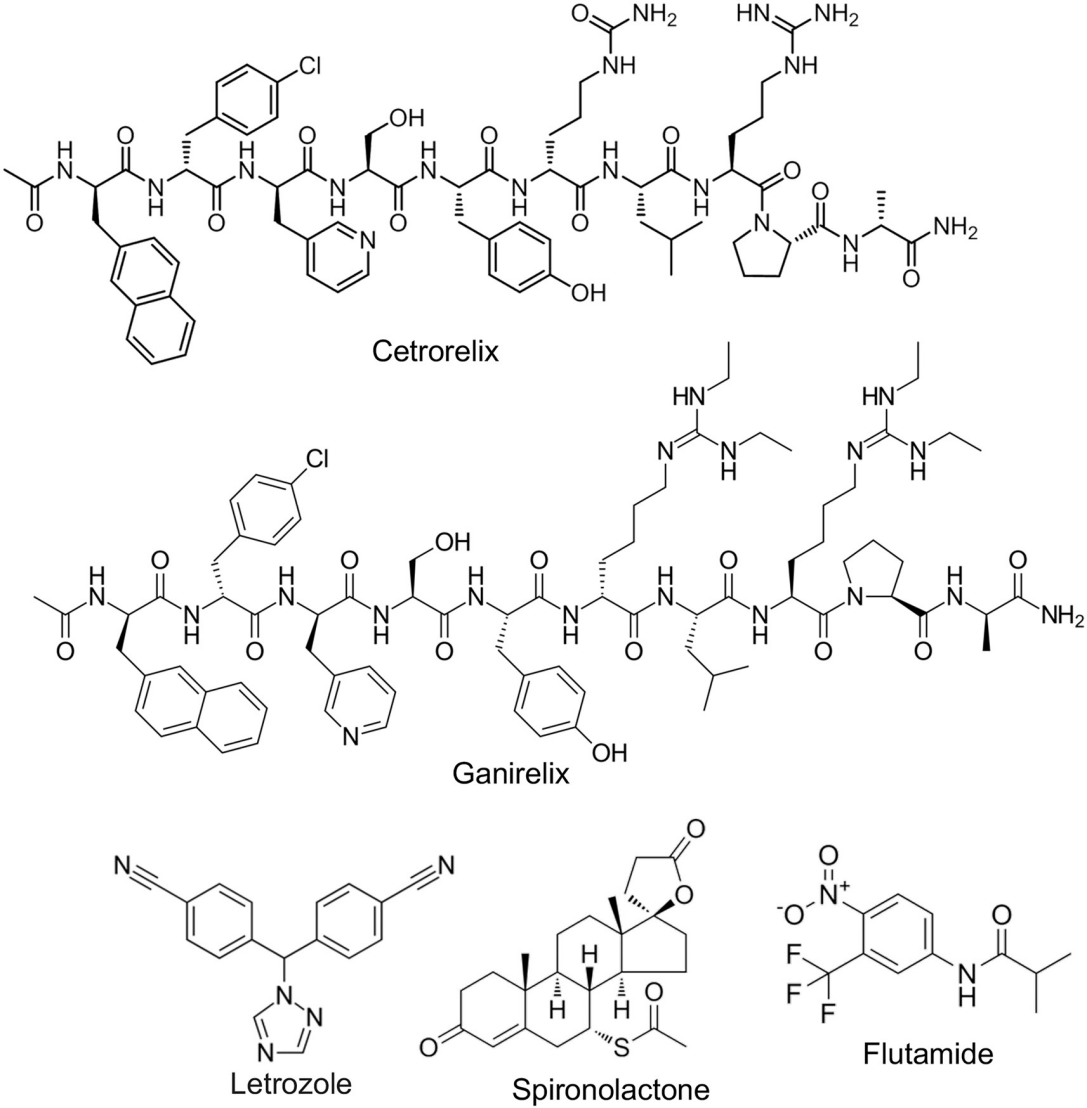
**Chemical structures of inhibitors of GnRH and aromatase and anti-androgens.** Chemical structures of cetrorelix and ganirelix (GnRH antagonists), letrozole (aromatase inhibitors), and spironolactone and flutamide (anti-androgens) are indicated. Abbreviation: GnRH: Gonadotropin-releasing hormone.

GnRH antagonists, such as cetrorelix and ganirelix (Figure 6), indirectly reduce AMH levels by suppressing gonadotropin secretion and lowering ovarian androgen production [73]. Androgens are known stimulators of AMH production in granulosa cells, creating a feedback loop that exacerbates the condition in PCOS [4]. By disrupting this loop, GnRH antagonists may lower serum AMH levels, thus enhancing FSH efficacy in follicular recruitment and maturation [73]. Clinical trials have indicated that GnRH antagonists are effective in restoring ovulation in women with PCOS, making them a valuable adjunct to AMH-targeted therapy [74].

### Aromatase inhibitors

Aromatase inhibitors, such as letrozole (Figure 6), are widely used in ovulation induction protocols for PCOS and can indirectly counteract the effects of elevated AMH levels [75]. By inhibiting the conversion of androgens to estrogens, these agents reduce estrogen-mediated negative feedback on the hypothalamic-pituitary axis, resulting in increased FSH secretion [75]. Elevated FSH levels can help overcome the inhibitory effects of AMH on follicular recruitment, thereby promoting the growth and maturation of dominant follicles. Letrozole has demonstrated superior efficacy compared to clomiphene citrate in inducing ovulation in women with PCOS, highlighting its potential to reverse AMH-mediated follicular arrest and improve fertility outcomes [75].

### Androgen-targeting therapies

Since androgens can stimulate AMH production in granulosa cells, targeting androgen levels may indirectly reduce AMH overexpression. Anti-androgens, such as spironolactone and flutamide, as well as androgen-lowering agents like oral contraceptives (Figure 6), can be used to modulate this axis [76]. Reducing androgen levels not only decreases AMH production but also improves follicular dynamics, offering a dual benefit in the management of PCOS [76].

## Future direction

AMH is a critical regulator of ovarian function and plays a central role in the pathophysiology of PCOS. Elevated AMH levels are a hallmark of PCOS and contribute to the disrupted follicular dynamics, anovulation, and hormonal imbalances characteristic of the condition [8, 10]. By inhibiting FSH sensitivity and suppressing primordial follicle activation, AMH perpetuates the follicular arrest commonly observed in PCOS. Additionally, AMH interacts with androgen signaling, exacerbating hyperandrogenism and creating a feedback loop that further impairs ovarian function [8, 10].

Despite significant advances, many aspects of AMH regulation and its role in PCOS remain incompletely understood. AMH expression is governed by a combination of transcriptional, post-transcriptional, and post-translational mechanisms. At the transcriptional level, key factors, such as GATA4, FOXL2, SF1, and WT1 have been identified as regulators of AMH gene expression [16–20]. However, the precise mechanisms through which these transcription factors interact with co-regulators and chromatin-modifying complexes to fine-tune AMH transcription remain largely unknown. Deciphering these complex regulatory networks is critical, as they may uncover novel pathways contributing to the elevated AMH levels observed in PCOS. Non-coding RNAs, including miRNAs and lncRNAs, also play essential roles in post-transcriptional regulation. Yet, the mechanisms underlying their dysregulation in PCOS are still poorly characterized [40–42]. In addition, post-translational modifications, such as proteolytic cleavage, glycosylation, and other covalent alterations are crucial for AMH maturation and function. These processes not only activate AMH but also affect its stability, bioactivity, and interaction with its receptor, AMHR2. The extent to which these modifications vary and influence AMH activity in the context of PCOS remains underexplored. A deeper understanding of these regulatory mechanisms is essential for fully elucidating AMH’s contribution to PCOS pathophysiology and may inform the development of targeted therapies.

The SMAD-dependent signaling pathway activated by AMH has been extensively studied for its role in regulating follicular development [51]. In PCOS, elevated AMH levels enhance SMAD1/5/8 signaling, which contributes to the suppression of follicular recruitment and growth. This dysregulation, in combination with altered FSH and androgen signaling, disrupts hormonal balance and impairs follicular maturation [51]. Despite these insights, the complete spectrum of genes and proteins regulated by AMH within these pathways remains incompletely characterized. Furthermore, AMH’s potential roles beyond the ovary—including possible systemic metabolic effects—are still poorly understood and warrant further investigation.

Given the promising preclinical findings, targeting AMH represents a potentially effective strategy for managing PCOS. Several therapeutic approaches—such as the development of AMHR2 antagonists, GnRH antagonists, and aromatase inhibitors—have been proposed to modulate AMH activity, restore FSH sensitivity, and promote normal folliculogenesis [69–72]. Additionally, combination therapies targeting both AMH and androgen signaling may offer synergistic benefits, improving both reproductive and metabolic outcomes. However, direct therapeutic targeting of AMH remains a nascent concept, with most strategies still in the preclinical phase. To fully establish AMH as a viable therapeutic target in PCOS, further research is required to develop more selective AMH inhibitors and to conduct robust clinical trials that rigorously assess their efficacy and safety. Importantly, PCOS is a heterogeneous disorder with significant phenotypic variability, including differences in hyperandrogenism, ovulatory function, and PCOM [1–4]. These variations, influenced by genetic, hormonal, and metabolic factors, can lead to divergent responses to treatment. Notably, AMH levels are often disproportionately elevated in specific PCOS phenotypes, particularly those characterized by severe anovulation and prominent PCOM [1–4]. This suggests that AMH-targeted therapies may not be universally applicable but could be most effective in selected subgroups. A personalized medicine approach—stratifying patients based on AMH levels, ovarian function, and clinical phenotype—may enhance therapeutic outcomes. For instance, individuals with high AMH expression and persistent anovulatory cycles may derive the greatest benefit from AMH or AMHR2 blockade. Future research should focus on defining these subpopulations more precisely and evaluating targeted interventions within well-characterized PCOS phenotype clusters.

Future research should prioritize a deeper exploration of the upstream and downstream signaling networks of AMH. Investigating how transcriptional regulators and epigenetic modifiers control AMH expression—alongside comprehensive profiling of miRNAs and lncRNAs in PCOS—could uncover novel therapeutic targets. Non-coding RNAs, including miRNAs and lncRNAs, have shown considerable promise in disease diagnosis and treatment [77]. While members of the miR-200 family, miR-140-3p, miR-155, and the lncRNA H19 have been implicated in the regulation of AMH expression, their specific roles in PCOS diagnosis and therapy remain to be fully defined [40–44]. In addition, exploring AMH’s systemic effects and its crosstalk with metabolic pathways may offer new insights into its broader role in PCOS pathophysiology. Stratifying patients based on AMH levels and hormonal profiles could facilitate the development of personalized treatment strategies, ultimately improving outcomes for individuals across the diverse spectrum of PCOS phenotypes.

## Conclusion

In conclusion, AMH is a pivotal factor in the pathogenesis of PCOS, functioning both as a biomarker and as a key regulator of ovarian dysfunction. While substantial progress has been made in elucidating its role, significant gaps remain in our understanding of the regulatory mechanisms and signaling pathways that control AMH expression and activity. Addressing these knowledge gaps will not only deepen our insight into PCOS pathophysiology but also support the development of targeted therapies aimed at improving reproductive and metabolic outcomes. With continued research, the full therapeutic potential of AMH-targeted interventions can be realized, paving the way for more effective and personalized treatments for individuals with PCOS.
